# Nasu-Hakola Disease

**DOI:** 10.5334/jbsr.2303

**Published:** 2020-11-30

**Authors:** Young Woo Sim, Sungjun Moon

**Affiliations:** 1 Yeungnam University Medical Center, KR; 2 Yeungnam University College of Medicine, KR

**Keywords:** Nasu-Hakola disease, Multiple bone cysts, Presenile dementia

## Abstract

**Teaching Point:** Nasu-Hakola disease (NHD) is characterized by multiple bone cysts in the appendicular skeleton and progressive presenile dementia.

## Case

**Figure 1:**
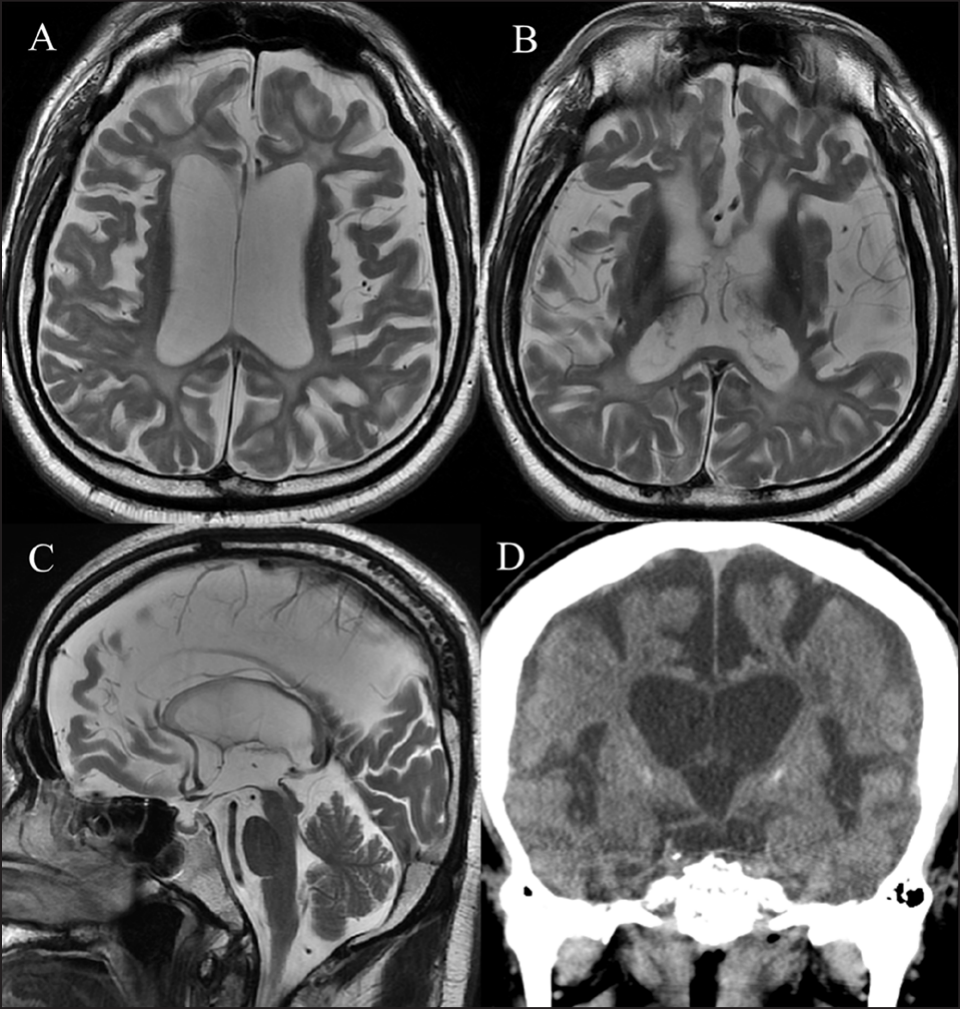


**Figure 2:**
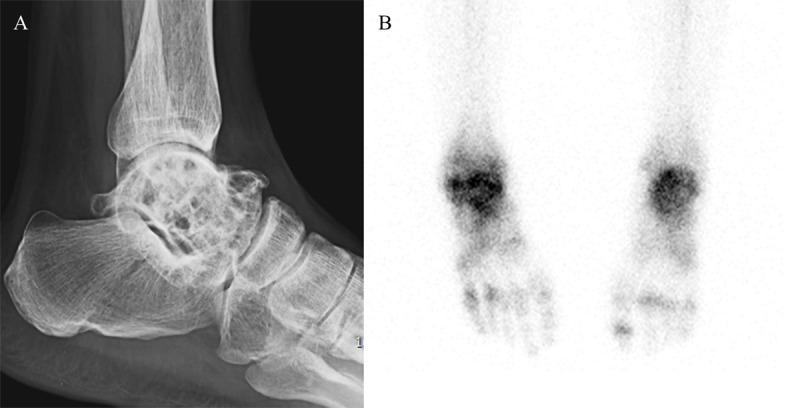


A 42-year-old man with diagnosed presenile dementia and Parkinsonism, was admitted for recurrent limb tonic-clonic movements. Electroencephalography showed diffuse slow activity. Brain MRI showed severe brain atrophy with severe white matter hyperintensity and basal ganglia atrophy with hypointensity, but relatively preserved occipital lobes and cerebellum (Figure 1A–C). Brain CT revealed bilateral calcifications in basal ganglia (Figure 1D). Previous pediatric charts revealed that the patient was born to non-consanguineous parents by vaginal delivery at home, and his development was apparently normal before entering school. However, his academic performance at school was poor and his IQ was rated at 80. At the age of late 20s, the patient was admitted to orthopedic department for pains of both ankles. Multiple bone cysts in both tali were visualized in foot radiographs, which symmetrically exhibited increased tracer uptake by bone scintigraphy (Figure 2A, B). The osseous lesions on microscopy consisted of convoluted lipid membrane structures filled with amorphous lipid. At the ages of early 30s, gait disturbance and memory impairment occurred and were progressively aggravated. Family members of the patient were clinically unremarkable.

## Comment

NHD is also well known as polycystic lipomembranous osteodysplasia with sclerosing leukoencephalopathy. NHD is a rare autosomal recessive disease, and clinically divided into four stages [1]. Latent stage is a period of early normal development, and osseous stage begins in the third decade and is accompanied by pathologic fractures caused by multiple bone cysts and trabecular loss. Early neurologic stage begins in the fourth decade and is characterized by frontal lobe syndrome and progressive dementia, and in late neurologic stage, global aphasia, loss of mobility and recurrent seizures occur. Affected patients progressively become vegetative state and pass away before the age of 50.

Multiple bone cysts in osseous stage are symmetrical in appendicular skeleton, and have fat-equivalent CT density and MR signal intensity. ^99^Tc methylene diphosphonate uptake is increased in these lesions. According to neurologic stage, variable degree of brain atrophy, especially in frontal lobes, are shown with variable extent of periventricular white matter hyperintensity on brain MRI. Bilateral calcifications in basal ganglia can be revealed on brain CT. PET and SPECT show decreased glucose metabolism and hypoperfusion in frontal and right basal ganglia, respectively [1].

Diagnosis is based on distinctive clinical and radiologic findings, and invasive biopsy is not necessary. Pathogenesis is suggested of osteoclast and microglia dysfunction according to TYROBP or TREM2 gene mutations [1]. TREM2/TYROBP is cell-surface receptor/adaptor signaling protein complex, which is expressed in myeloid cells such as microglia and osteoclasts. Molecular study can aid early diagnosis in the latent stage and familial genetic consultation. Wide awareness of this disease entity can prevent unnecessary procedure.
